# Replicating File Segments between Multi-Cloud Nodes in a Smart City: A Machine Learning Approach

**DOI:** 10.3390/s23104639

**Published:** 2023-05-10

**Authors:** Nour Mostafa, Yehia Kotb, Zakwan Al-Arnaout, Samer Alabed, Ahmed Younes Shdefat

**Affiliations:** 1 College of Engineering and Technology, American University of the Middle East, Egaila 54200, Kuwait; yehia.kotb@aum.edu.kw (Y.K.); zakwan.alarnaout@aum.edu.kw (Z.A.-A.); ahmed.shdefat@aum.edu.kw (A.Y.S.); 2 Biomedical Engineering Department, School of Applied Medical Sciences, German Jordanian University, Amman 11180, Jordan; samer.alabed@gju.edu.jo

**Keywords:** multi-cloud, edge servers, internet of things (IoT), data replication, partial replication, system soundness, formal methods, regression, machine learning

## Abstract

The design and management of smart cities and the IoT is a multidimensional problem. One of those dimensions is cloud and edge computing management. Due to the complexity of the problem, resource sharing is one of the vital and major components that when enhanced, the performance of the whole system is enhanced. Research in data access and storage in multi-clouds and edge servers can broadly be classified to data centers and computational centers. The main aim of data centers is to provide services for accessing, sharing and modifying large databases. On the other hand, the aim of computational centers is to provide services for sharing resources. Present and future distributed applications need to deal with very large multi-petabyte datasets and increasing numbers of associated users and resources. The emergence of IoT-based, multi-cloud systems as a potential solution for large computational and data management problems has initiated significant research activity in the area. Due to the considerable increase in data production and data sharing within scientific communities, the need for improvements in data access and data availability cannot be overlooked. It can be argued that the current approaches of large dataset management do not solve all problems associated with big data and large datasets. The heterogeneity and veracity of big data require careful management. One of the issues for managing big data in a multi-cloud system is the scalability and expendability of the system under consideration. Data replication ensures server load balancing, data availability and improved data access time. The proposed model minimises the cost of data services through minimising a cost function that takes storage cost, host access cost and communication cost into consideration. The relative weights between different components is learned through history and it is different from a cloud to another. The model ensures that data are replicated in a way that increases availability while at the same time decreasing the overall cost of data storage and access time. Using the proposed model avoids the overheads of the traditional full replication techniques. The proposed model is mathematically proven to be sound and valid.

## 1. Introduction

There is no a unique agreed-upon definition for a smart city [1]. As for any big concept, definitions are set based on the objective of using the concept of smart cities. Due to the complexity of smart city concepts, many definitions are being used among the research community. While researchers adopt different definitions for smart city for different objectives, they all agree on the components that form a smart city [2]. Some of these components are cloud computing, edge computing, communication infrastructure, and data access [3]. Cloud computing is an approach that ensures resource sharing among users with minimal management. Resources could be networks, servers, storage, applications, services or data [4]. Cloud resource management is a research field that has been studied for the last two decades and most of its problems are being solved. Two problems are still hot topics: edge computing [5] and multi-cloud computing [6]. Edge computing is the field that is concerned with policies and techniques to migrate some of the computations and decision making from cloud to edge servers in order to minimise communication cost and to reduce some of the load that is handled by cloud servers so that cloud servers are left with tasks that cannot be performed on edge servers. Since the majority of edge servers are limited in resources, edge computing is still striving for contributions to solve many of its problems [5]. Multi-cloud computing is the approach of having multi-clouds cooperating together as a cooperative distributed system to solve problems that cannot be solved in a single cloud which are defined in [6,7].

Scientific applications typically involve high-throughput experiments, such as satellite surveys [8], supercomputer simulations [9,10] and sensor networks [11], which generate petabytes of scientific data, in addition to the massive data generated by internet every second. For example, the production of data within a radiology department in a hospital in an industrialised country such as the United States or Western Europe is on the order of 10 terabytes a year [12]. In European countries, the total data produced are in the order of petabytes per year and the total medical data of Europe or the United States can be estimated at thousands of petabytes. Furthermore, at present the largest astronomy database is around 20 terabytes per night. Nowadays, data generation is estimated 44 times more than that in 2009 [13]. At present the healthcare data worldwide are in terabytes (10^12^ bytes), and it is expected in the future to be increased to zetaabyte (10^21^ bytes) or yottabyte (10^24^ bytes) [14]. Storing, accessing and analysing such huge data sets requires a means of efficiently organising, handling and manipulating high-volume data. Systems to address these fundamental issues are a focus of current research. As mentioned earlier, cloud, edge servers and IoT systems must have the capability to deal with the huge number of resources and users at the same time. Increased size sometimes presents the issue of performance degradation [15]. Therefore, such systems should be able to support adaptability, scalability and extensibility to avoid such degradation. The proposed partial replication algorithm allows users to replicate parts of files instead of replicating the full file. Hence, when a user submits a task that requires one or more files or segment of a file to be executed, the replica service uses the new portioning algorithm to divide a file into segments and transfer and save the segment(s) required by the task to the user’s resources. The new replication system is treated as an optimisation solution that minimises the sum of the data access costs and achieves good system utilisation. This paper defines a multi-cloud edge hybrid system where resources are being shared and nodes cooperate together to ensure the availability of data with a minimum overall cost as will be seen later in this paper. The goal of a data cloud is to provide services for accessing, sharing and modifying large databases [16]. However, as the number of resources contributing to the cloud, edge servers and IoT grows, the complexity of managing these resources increases [17]. This complexity leads to larger databases and longer delays in task execution due to the need to locate multiple files stored in different sites [18]. To address this issue, intelligent management of terabyte data transfer over wide area networks is necessary to cope with current and future data [19,20].

There are a number of aims of the work presented here. One is to build the base model which supports task submission (i.e., requests of data files from single and multiple users). The second goal is to consider existing techniques and retain the “best” of these approaches. The third goal is to develop a partial replicas technique and investigate its effect on the system. The proposed system can access the relevant segment of a replica in a minimum response time, for given task under execution. The new replication system is treated as an optimisation problem that minimises the sum of the data access costs and achieves good system utilisation. The performance of the new system is better than a similar system using full dynamic replication. The partial replication algorithm has a significant impact on system performance in particular the operation of accessing distributed files enabling overall tasks turnaround times and resource consumption to be decreased. In this paper, an algorithm is proposed to find the best replication candidate with the minimum routing cost. The best candidate is chosen so that the cost of the whole system is minimised. A closed-form theorem that sets the constraints of system soundness is proposed and proved.

This paper is organised as follows: Section 1 introduces the paper and clarifies the different topics that are discussed throughout the paper. Section 2 defines the problem that is solved in this paper.

Section 3 presents the literature and previous work that contributed in solving similar problems. Section 4 presents the reference model of full replication scheme. Section 5 demonstrates the proposed model and its components. Section 6 demonstrates the file replication cost and its components. Section 7 explains the simulation and shows the results of different experiments. Finally, Section 8 concludes the work that is performed and summarises the results obtained from simulation.

## 2. Problem Statement

Increasing the performance of cloud responses to user requests is a major requirement for a better and smarter city [21]. The problem that this paper is solving can be summarised as follows: having a multi-cloud system with clouds that are interconnected and with multiple edge servers connected to clouds and/or other edge servers directly or indirectly, and with some clouds or edge servers that have file resources, it is required to replicate parts of files as needed so that they are accessible and reachable with minimum cost to edge servers. As will be seen in Section 5, the cost has different components with different weights depending on the node whether this node is a cloud or an edge server. Mathematically, the problem is defined as follows:

The system S is defined as:

(1) S=〈C,E,F,Ξ〉

where:

(2) $$C=\{c_{1},c_{2},c_{3},\ldots ,c_{n}\}$$

where C is the set of clouds in a multi-cloud system, and n=∥C∥ and $c_{i}$ is cloud *i* where 1≤i≤n and:

(3) $$E=\{e_{1},e_{2},e_{3},\ldots ,e_{m}\}$$

where E is the set of edge servers connected to this multi-cloud system and m=∥E∥ and $e_{j}$ is edge server *j* where 1≤j≤m and:

(4) $$F=\{f_{1},f_{2},f_{3},\ldots ,f_{l}\}$$

where F is the set of files that are being shared in the multi-cloud system, l=∥F∥ and $f_{k}$ is distinct file *k* where 1≤k≤l and Ξ is the topology that describes the inter-connectivity of the system. It is required to replicate segments of the shared files so that the overall cost of file sharing is minimum. The cost of replicating a file segment *s* so that certain node $n_{d}$ accesses it is composed of:

1. The transmission cost from the source node $n_{i}$ to replica node $n_{j}$
2. The hosting cost of the replica $n_{j}$
3. The transmission cost from replica $n_{j}$ to $n_{d}$

Total cost η is defined to be:

(5) $$\eta =\beta_{1}\pi +\beta_{2}\rho +\beta_{3}\tau$$

where π is the data hosting cost, ρ is the load on the hosting node, and τ is the cost of data transfer delay. The difference between π and ρ is that π represents the cost of replicating certain file or file segment in certain node whereas ρ is the cost of raising load on certain node. Every node has its own weighting for π, ρ and τ, and therefore every node learns $\beta_{1},\beta_{2}\;\mathrm{and}\;\beta_{3}$ from historic data. The final objective is to obtain cost where cost is defined to be:

(6) cost=Minimizeη(c∈C)

Note that the unit costs for every independent variable in Equation (5) is determined by the host and the weights are learned through historical data. When replicating a file f∈F, the cost of data transfer and hosting depends on how big a file is. This means that replicating parts of the file could minimise both hosting and data transfer costs.

## 3. Related Work

The authors in [22] proposed a replica creation and selection model using the replica creation based on access tendency (DRC-AT), and the replica selection based on response time (DRS-RT). The proposed model calculates the size of the file access tendency, which is used for the replica creation decision, and the user’s request time is analysed and evaluated to select the best node that contains the requested data by the tasks submitted by the user. The authors in [23] proposed a replica replacement algorithm which predicts the future usage of the replica using the weight value and normalisation by calculating the three factors, cost of replica, frequency and number of requests. The proposed model uses a prediction function that computes the relative worth of replica using the history of the file access, then it determines the priority of the replica placement. The authors in [24] proposed an algorithm for accessing a subset of a spatial replica using a greedy approach which chooses replica subsets which allow fast data access to maximise performance. Storing the replica subset in descending order by calculating the goodness value of each subset. The proposed model aiming to provide load balancing by taking into consideration the hardware performance, filesystem and file storage prefetching. The authors in [25] investigate the effect of replication role in Distributed Transactional Memory (DMT) and comparing a full replication scheme and partial replication approaches. The study shows that the partial replication approaches have an obvious improvement in terms of scalability by reducing the amount of transferred and stored data at each node. To ensure data consistency, the study mentioned several existing techniques such as single copy model [26,27], Distributed Multiversioning (DMV) system model [28], History-based multiversioning [29] and Clock Validation [30,31]. This study also investigates the latency issues in partial replication in the context of DMT.

The authors in [32] proposed a nonlinear integer programming model for data replication in cloud storage aiming to achieve a low cost and high availability. The proposed low-cost failure resilient replication scheme is handling both non-correlated and correlated machine failure. The proposed model shows an improvement in data availability and replica consistency cost compared to Random Replication (RR) [33], Copyset Replication [33] and Replication Degree Customisation (RDC) [34]. The proposed scheme assigns a portion replica for each data object and the popularity of the replica is considered by handling the aforementioned problems. The popularity of any two data objects are analysed and compared to reduce the replication cost. The authors in [35] investigate the use of object storage system in the cloud storage environment to store and retrieve unstructured data in cloud computing environment. The object storage system process data in a different way compared to modern storage system which process data as objects instead of blocks and files. All data types can be stored in an object, such as records, file, database, medical record, video, audio and images, or it can be used to store only an entire type of data. This paper analysed the different types of storage systems used in cloud computing environment, the authors claiming that the object storage is the most suitable storage system for unstructured static data to be used in cloud storage environment due to its scalability, flexibility and security features.

The authors in [36] proposed a replica placement model based on evaluating the comprehensive performance value of the node. The proposed model used the Hadoop Distributed File System (HDFS) as the base model to be improved which divide the data into small blocks as the basic storage unit to be stored on different distributed nodes. The proposed model calculates the weight of the evaluation value of set of indicators such as the evaluation values of memory size, disk space, CPU and read-write speed of the disk, then the best set of replica nodes will be selected using the comprehensive evaluation value.

The authors in [37] proposed a hybrid data replication system for edge servers and cloud infrastructure which reduce the latency perceived by read and update operations by locating the replica near the end user. This paper proposed a replica convergence algorithm which keeps replicas in both the cloud and the edge server by combining Conflict-free Replicated Data Types (CRDTs) and Operational Transformation (OT) to achieve a consistency model. The proposed model is suitable for the applications based on microservices as it follows the hierarchical architecture of a master replica which broadcasts updates received from a particular replica. The authors in [38] proposed a partial storage strategy for cloud data centers by partitioning the dual direction download of files from different cloud storage. The proposed model introduced an improvement to the dual directional algorithm (DDFTP) which is used as a file retrieval from the cloud server. This algorithm divides the data into blocks. Then each block will be assigned to two cloud servers based on the download history of these blocks which in turn will be used to download the data from opposite directions. The download process will be parallel by handling the assignment of the forward and backward in the block, and the proposed portioning technique removes some blocks from each replica based on the history download experience. The proposed model did not take into consideration many factors such as memory speed, size, server failure and fault tolerance issues which affect the effectiveness and efficiency of the system.

In [39] the authors proposed an improved cache utilisation model which identifies user hot-spots, where fit clients are selected for partial caching. The proposed model uses a location tracking and prediction method to identify hot-spot locations to be used later by service’s subscribers. The client nodes in the proposed model works as service providers and coupled with subscribers’ latency. The authors in [40] proposed a collaborative fog-to-fog communication algorithm which allow fogs to communicate with each other to process incoming tasks. In this proposed model, a threshold is set for maximum delay allowed. If the delay of the assigned fog reaches the threshold, then the fog will check the list of available candidate fogs which can service it and delegate the task to this candidate. In [41] the authors proposed a fog resource selection algorithm (FResS) which enables automated fog selection and allocation, the performance data of each fog are stored in standard format as execution logs. For an incoming job to be executed, these logs will be used for predicting its run-time to have real time estimate for best fog selection.In [42] the authors proposed a neural network prediction model to predict replica locations using the files’ access profile. The run-time prediction model is meant to generate file location predictions for incoming tasks using historical executions. It utilises data clustering techniques to separate related tasks from the history and generates a prediction of a file’s location using a mean predictor. On the other hand, the proposed model takes some factors into consideration to minimise a cost function using storage cost, host access cost and communication cost to achieve the minimum cost of the data service. In addition, the proposed model calculates the cost difference between replicating a whole file and a segment of a file on a certain node.

## 4. Reference Models-Full Replication

Replica management service is responsible for initiating data replication when needed, in addition to creating or deleting copies of files, or replicas, stored in a specific storage system. The design of the replica management service is modular, with several independent services, i.e., task scheduling, resource discovery, etc. and interacting via the Replica Manager (RM). The RM coordinates the interaction between all components of the replica management system i.e., if a new storage location offers better performance and availability for access to or from a particular location, then the replica manager will create a replica at the new location [43]. In addition, cloud, edge server and IoT environments are highly dynamic whereby the resource’s availability and performance change constantly. Therefore the replica management service is responsible for discovering new replicas which may be added to or deleted from different locations.

Replica creation and/or selection is the second representative of the high level services provided by a cloud storage. Cloud storage technology was developed to share data between different organisations across distributed geographical locations in an efficient way. Cloud storage uses a data replication technique to move data closer to users thereby improving data access performance [44]. The replica selection service is responsible for finding the best replica that will minimise the transfer time, i.e., finding the nearest copy to the user, so the selection process uses the absolute performance technique (e.g., speed, cost or security). Cloud Information Services (CIS) is a cloud service which provides information about network performance. The replica selection service uses this information and the information provided by metadata repository (i.e., file size, location, etc.) to determine which storage will yield the fastest data access [45].

As previously mentioned, at present, the size of the data that need to be accessed daily on the cloud, edge servers and IoT is in the order of thousands of petabytes and by 2025 the amount of data generated globally will reach 175 ZB (zettabytes), the IoT applications and devices will be the main source of this amount [46,47]. Ensuring efficient access to such vast and widely distributed data is a serious challenge to network and cloud designers. Replication is one such widely accepted technique in distributed environment by storing the data at more than one site. If a data site fails, the system can still continue to operate using replicated data, increasing availability and fault tolerance. At the same time, as the data are stored at multiple sites, the request can find the data close to the site where the request originated, thus increasing the efficiency of the system, lowering bandwidth consumption and improving scalability of the overall system [48,49].

**Figure 1:**
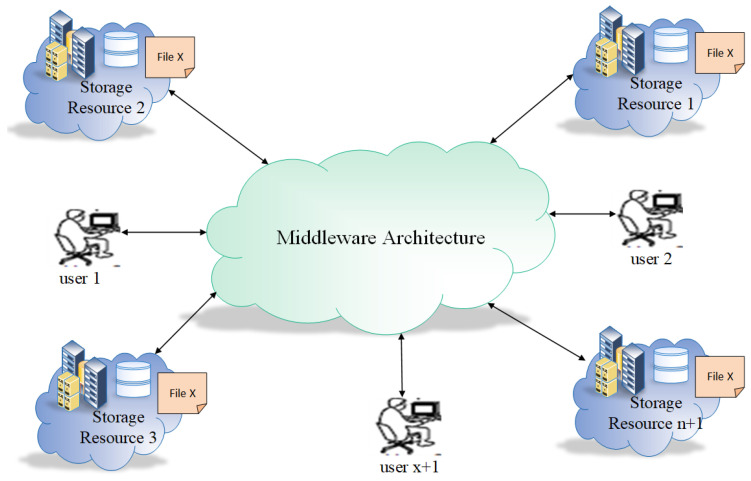
Replicas environment.

In the following Figure 1 replicas environment is presented in a simple manner. Storage resource 1, storage resource 2, storage resource 3, storage resource n, are distributed resource locations and connected through a middleware infrastructure. A file, i.e., File X used to hold the data, is stored in storage resource 2 and all other resources replicates File X. In this example the benefits of replication are clear (as storage resource 1 and storage resource 3 are close to the user compared to storage resource 2, where the file was originally stored). The access cost of files can be decreased, thus improving the performance and availability even if three out of four storage resources are down.

## 5. Using Machine Learning for Partial Replication and Selecting the Candidate Node

Multivariable regression is a statistical method used in machine learning to model the relationships between multiple independent variables and a dependent variable. It is a powerful tool for analysing complex datasets and it is used in a wide range of applications, including finance, healthcare, marketing and social sciences. In simple linear regression, a single independent variable is used to predict the value of a dependent variable. However, in many real-world scenarios, a single independent variable may not be enough to fully explain the variability in the dependent variable. For example, in predicting the price of a house, factors such as location, size, number of rooms and age of the house may all be important, and a multivariable regression model can capture the influence of each of these factors on the house price.

Multivariable regression models can take different forms, including linear regression, logistic regression and polynomial regression. Linear regression is the most commonly used form and involves finding a line that best fits the data points by minimising the sum of the squared errors between the predicted values and the actual values. Logistic regression is used when the dependent variable is binary, such as predicting whether a customer will buy a product or not. Polynomial regression is used when the relationship between the independent and dependent variables is nonlinear.

One of the advantages of multivariable regression is its ability to control confounding variables. Confounding variables are variables that are correlated with both the independent and dependent variables and can lead to spurious associations. Multivariable regression allows the inclusion of these variables in the model, thereby providing more accurate estimates for the effects of the independent variables on the dependent variable.

Multivariable regression is a valuable tool for data analysis and can provide insights into the relationships between variables and help in making predictions about future outcomes. However, it is important to ensure that the assumptions of the model are met and the model is not overfitting the data, as this can lead to inaccurate predictions. Careful data cleaning and feature selection are essential to ensure that the model is robust and generalisable to new data.

A multivariable regression can be represented by a general equation, given by:

(7) $$Y=\beta_{0}+\beta_{1}X_{1}+\beta_{2}X_{2}+\ldots +\beta_{n}X_{n}$$

Here, Y is the dependent variable and $X_{i}$ represents the *i*th independent variable. The coefficients $\beta_{i}$ are the weights assigned to each independent variable, indicating the strength of its relationship with the dependent variable.

The coefficient $\beta_{0}$ is the intercept, which represents the expected value of the dependent variable when all independent variables are zero. It is also known as the constant term or the error term, as it captures the overall effect of any unmeasured factors or errors in the model.

In this paper, the aim is to select the most efficient server for replication based on several parameters. Since the task’s execution time depends on multiple variables, such as the server’s processing power, memory size and network bandwidth, we employ multivariable linear regression. This technique allows us to create a model that can predict the duration required to execute a task on each server, given the various parameters.

To train our model, we use historical data that include information about previous tasks and the servers used to complete them, as well as the corresponding execution times. Once the model is trained, we can use it to predict the duration required to execute a new task on each available server.

The model we use for prediction is represented by Equation (54). This equation takes into account the various parameters that influence the task’s execution time, such as the file size, disk size and the server’s processing power, memory size and network bandwidth. By substituting in the relevant values for each server, we can determine which server offers the shortest duration to execute the task that accesses the replicated file segment. Utilising multivariable linear regression and Equation (54) enables us to select the most efficient server for a given task based on multiple parameters, leading to a significant reduction in task execution time and increased system performance.

The topology Ξ is presented in Equation (1) where it represents the interconnections between nodes in a system. These nodes are either clouds or edge servers and the possible alternative for node communication is as follows:

1. Communication between two clouds,
2. Communication between two edge servers,
3. Communication between a cloud and an edge server.

Note that in this paper it is assumed that graphs are bi-directional. In other words, the communication cost from node $n_{1}$ to node $n_{2}$ is the same code from node $n_{2}$ to node $n_{1}$. As mentioned above, topology Ξ represents connection among all nodes regardless whether they are clouds or edge servers. Topology Ξ is represented as follows:

(8) Ξ≡〈Φ,Ω,Ψ〉

Sub-Topology Φ represents the interconnections among different clouds. Φ is represented as follows:

(9) Φ≡C×C

which is the cross product between the set of clouds and itself, this given an n×n square matrix and n=∥C∥. Sub-Topology matrix Φ is filled as follows:

(10) $$\Phi \left(i,j\right)=\left\{\begin{array}{cc} \Phi (i,j)=1 & \mathrm{if}\;i=j\\ \Phi (j,i)=1 & \mathrm{if}\;c_{i}\;\mathrm{and}\;c_{j}\;\mathrm{are}\;\mathrm{connected}.\\ \Phi (j,i)=0 & \mathrm{if}\;c_{i}\;\mathrm{and}\;c_{j}\;\mathrm{are}\;\mathrm{not}\;\mathrm{connected}. \end{array}\right.$$

Equation (10) describes how matrix Φ is filled, the first case is when it is a diagonal element of the matrix and in this case, the element is always 1 since every cloud is reachable from itself. The second case is when two clouds $c_{i}\;\mathrm{and}\;c_{j}$ are directly connected together where the element is set to 1. The third and final case is when the element is not a diagonal and two clouds intersecting at this element where there is no direct connection that links the two of them, that is when the element is set to 0. The second sub-topology is Ω which represents the connections between edges. This is an m×m square matrix and m=∥E∥. Sub-Topology Ω can be formulated as follows:

(11) Ω≡E×E

Sub-Topology matrix Φ is filled as follows:

(12) $$\Omega \left(i,j\right)=\left\{\begin{array}{cc} \Omega (i,j)=1 & \mathrm{if}\;i=j\\ \Omega (j,i)=1 & \mathrm{if}\;e_{i}\;\mathrm{and}\;e_{j}\;\mathrm{are}\;\mathrm{connected}\\ \Omega (j,i)=0 & \mathrm{if}\;e_{i}\;\mathrm{and}\;e_{j}\;\mathrm{are}\;\mathrm{not}\;\mathrm{connected} \end{array}\right.$$

Equation (12) describes how matrix Ω is filled. The first case is when it is a diagonal element of the matrix and, in this case, that element is always 1 since every edge is reachable from itself. The second case is when two edges $e_{i}\;\mathrm{and}\;e_{j}$ are directly connected together and that is when the element is set to 1. The third and final case is when the element is not a diagonal and two edge servers intersecting at this element where there is no direct connection that links the two of them; that is when the element is set to 0. The third sub-topology is Ψ which represents the connections between edge servers and clouds, this is an n×m matrix where n=∥C∥ and m=∥E∥. Sub-Topology Ψ is formulated as follows:

(13) Ψ=C×E∪E×C

Note here that, since the topology is represented as a bipartite graph, the union of the equation is necessary since the order here matters as it defines the flow direction. Sub-Topology matrix Ψ is filled as follows:

(14) $$\Psi \left(i,j\right)=\left\{\begin{array}{cc} \Psi (j,i)=1 & \mathrm{if}\;c_{i}\;\mathrm{and}\;e_{j}\;\mathrm{are}\;\mathrm{connected}.\\ \Psi (j,i)=0 & \mathrm{if}\;c_{i}\;\mathrm{and}\;e_{j}\;\mathrm{are}\;\mathrm{not}\;\mathrm{connected}. \end{array}\right.$$

Equation (14) describes how matrix Ψ is filled. The first case is when a cloud $c_{i}$ and an edge server $e_{j}$ are directly connected together and that is when the element is set to 1. The second and final case is when $c_{i}$ and an edge server $e_{j}$ are not connected and that is when the element is set to 0. The overall topology Ξ is formulated as follows:

(15) $$\Xi =\left[\begin{array}{cc} \Phi & \Psi \\ \Psi^{T} & \Omega \end{array}\right]$$

where $\Psi^{T}$ is the transpose of matrix Ψ, and Ξ is an (n+m)×(n+m) square matrix where n×m matrix where n=∥C∥=∥Φ∥ and m=∥E∥=∥Ω∥.

### 5.1. Example of Topology Formulation

**Figure 2:**
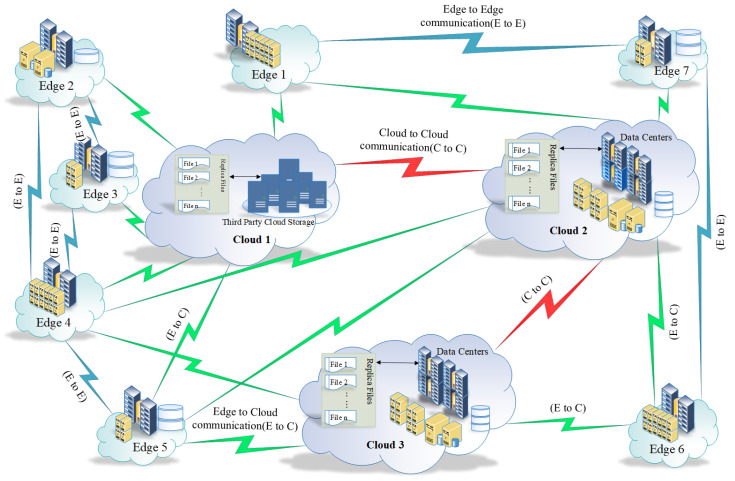
Topology Ξ for system S.

As shown in Figure 2, edge servers are used to provide content and services closer to the user, while cloud servers are used to store and manage data and applications in a remote location. Edge servers are typically located at the edge of the network, while cloud servers are located in a centralised data center. Edge servers are used to reduce latency and improve performance, while cloud servers are used to provide scalability and cost savings. Edge nodes can serve as intermediate nodes between two or more clouds in communication. In a distributed cloud architecture, there may be multiple cloud nodes that are geographically distributed across different regions or even countries. In such a scenario, it may be inefficient to transmit data directly between the clouds, especially if the data need to travel long distances or cross international borders. In this case, edge nodes can be used as intermediate nodes between the clouds. An edge node located near the source cloud can receive the data and process them locally, before transmitting them to another edge node located near the destination cloud. This approach can help to reduce the overall latency and bandwidth requirements of the communication. In addition, edge nodes can also perform other functions such as caching frequently accessed data, filtering or preprocessing data before they are transmitted to the cloud and providing additional security measures such as encryption and access control [50,51]. To demonstrate the idea, consider the topology shown in Figure 2 which shows a system S with three clouds and eight edges.

Figure 2 demonstrates the problem to be solved, where users are trying to access some file segments on the cloud and because of the constraints that are discussed and defined in Section 2, the cost of accessing those files is high. The system shown in Figure 2 is described as follows:

From Equations (9) and (10), we can see that Φ for the system shown in Figure 2 is:

(16) $$\Phi =\left[\begin{array}{ccc} 1 & 1 & 0\\ 1 & 1 & 1\\ 0 & 1 & 1 \end{array}\right]$$

Equation (16) shows that $c_{1}$ and $c_{2}$ are directly connected, and $c_{2}$ and $c_{3}$ are also directly connected. Topology matrices can be seen as reachability matrices, which means that it tells which node is reachable from another. The matrix represents the reachability of clouds from other clouds and since any cloud is reachable from itself, the diagonal is always 1. Since the graph is actually bi-directional one can see that the matrix is a symmetric matrix. From Equations (11) and (12) we can see that Ω for the system shown in Figure 2 is:

(17) $$\Omega =\left[\begin{array}{ccccccc} 1 & 0 & 0 & 0 & 0 & 0 & 1\\ 0 & 1 & 1 & 1 & 0 & 0 & 0\\ 0 & 1 & 1 & 1 & 0 & 0 & 0\\ 0 & 1 & 1 & 1 & 1 & 0 & 0\\ 0 & 0 & 0 & 1 & 1 & 0 & 0\\ 0 & 0 & 0 & 0 & 0 & 1 & 1\\ 1 & 0 & 0 & 0 & 0 & 1 & 1 \end{array}\right]$$

Equation (17) shows that $e_{1}$ is directly connected with $e_{7}$. Furthermore, $e_{2}$ is directly connected with both $e_{3}$ and $e_{4}$. $e_{3}$ is directly connected with both $e_{2}$ and $e_{4}$. $e_{4}$ is directly connected with $e_{2},e_{3}$ and $e_{5}$. $e_{5}$ is directly connected with $e_{4}$. $e_{6}$ is directly connected with $e_{7}$. Finally, $e_{7}$ is directly connected with both $e_{1}$ and $e_{6}$.

(18) $$C=\{c_{1},c_{2},c_{3}\}$$

(19) $$E=\{e_{1},e_{2},e_{3},e_{4},e_{5},e_{6},e_{7},e_{8}\}$$

The matrix represents the reachability of edge servers from edge servers and, since any edge server is reachable by itself, the diagonal is always 1. Since the graph is actually bi-directional one can see that the matrix is a symmetric matrix. From Equations (13) and (14) we can see that Ψ for the system shown in Figure 2 is:

(20) $$\Psi =\left[\begin{array}{ccccccc} 1 & 1 & 1 & 1 & 1 & 0 & 0\\ 1 & 0 & 0 & 1 & 1 & 1 & 1\\ 0 & 0 & 0 & 1 & 1 & 1 & 0 \end{array}\right]$$

Equation (20) shows that $c_{1}$ is connected with $e_{1},e_{2},e_{3},e_{4}$ and $e_{5}$. $c_{2}$ is connected with $e_{1},e_{4},e_{5},e_{6}$ and $e_{7}$. Finally, $c_{3}$ is connected with $e_{4},e_{5}$ and $e_{6}$. The matrix represents the reachability of clouds from edge servers or edge servers from clouds. Moreover the matrix does not have to be a square matrix since the number of clouds and edge servers do not have to be the same. From Equations (15)–(17) and (20) we can see that the overall topology Ξ is represented as follows:

(21) $$\Xi =\left[\begin{array}{cccccccccc} 1 & 1 & 0\; & \;1 & 1 & 1 & 1 & 1 & 0 & 0\\ 1 & 1 & 1 & \;1 & 0 & 0 & 1 & 1 & 1 & 1\\ 0 & 1 & 1 & \;0 & 0 & 0 & 1 & 1 & 1 & 0\\ 1 & 1 & 0 & \;1 & 0 & 0 & 0 & 0 & 0 & 1\\ 1 & 0 & 0 & \;0 & 1 & 1 & 1 & 0 & 0 & 0\\ 1 & 0 & 0 & \;0 & 1 & 1 & 1 & 0 & 0 & 0\\ 1 & 1 & 1 & \;0 & 1 & 1 & 1 & 1 & 0 & 0\\ 1 & 1 & 1 & \;0 & 0 & 0 & 1 & 1 & 0 & 0\\ 0 & 1 & 1 & \;0 & 0 & 0 & 0 & 0 & 1 & 1\\ 0 & 1 & 0 & \;1 & 0 & 0 & 0 & 0 & 1 & 1 \end{array}\right]$$

The top left corner of the matrix in Equation (21) is the matrix in Equation (16). The top right corner of the matrix in Equation (21) is the matrix in Equation (20). The bottom left corner of matrix in Equation (21) is the transpose of the matrix in Equation (20). Finally, the bottom right corner of the matrix in Equation (21) is the matrix in Equation (17). Removing separation lines is given in Equation (22).

(22) $$\Xi =\left[\begin{array}{cccccccccc} 1 & 1 & 0 & 1 & 1 & 1 & 1 & 1 & 0 & 0\\ 1 & 1 & 1 & 1 & 0 & 0 & 1 & 1 & 1 & 1\\ 0 & 1 & 1 & 0 & 0 & 0 & 1 & 1 & 1 & 0\\ 1 & 1 & 0 & 1 & 0 & 0 & 0 & 0 & 0 & 1\\ 1 & 0 & 0 & 0 & 1 & 1 & 1 & 0 & 0 & 0\\ 1 & 0 & 0 & 0 & 1 & 1 & 1 & 0 & 0 & 0\\ 1 & 1 & 1 & 0 & 1 & 1 & 1 & 1 & 0 & 0\\ 1 & 1 & 1 & 0 & 0 & 0 & 1 & 1 & 0 & 0\\ 0 & 1 & 1 & 0 & 0 & 0 & 0 & 0 & 1 & 1\\ 0 & 1 & 0 & 1 & 0 & 0 & 0 & 0 & 1 & 1 \end{array}\right]$$

Topology Ξ in Equation (22) is represented as a square matrix with size of (n+m)×(n+m) where n=∥C∥=3 and m=∥E=7.

### 5.2. Topology Operator

Operator ⟷ is a proposed mathematical binary operator that, when applied, finds the path from two nodes. x⟷y gives all possible paths between *x* and *y*. The convention that is followed when using this operator is as follows:

1. x⟷y means the path when *x* and *y* are directly connected. We call this 0-degree path.
2. $x\overset{1}{⟷}y$ means the set of all paths when *x* and *y* are indirectly connected and there is only one node in the middle. We call paths that belong to this set 1st degree paths.
3. $x\overset{1}{\underset{z}{⇄}}y$ means the set of all paths when *x* and *y* are indirectly connected with one node in the middle and that node is *z*.
4. $x\overset{n}{\underset{z}{⇄}}y$ means the set of all paths when *x* and *y* are indirectly connected with n node in the middle and z is in those nodes. We call paths that belong to this set $n^{th}$ degree paths.
5. $x\overset{n}{\underset{a,b,c,\ldots ,z}{⇄}}y$ means the set of all paths when *x* and *y* are indirectly connected with n node in the middle and a,b,c,…,z are in those nodes. Note that x,y∉a,b,c,…,z.

### 5.3. Proof of Soundness

This section proves the formal soundness of operator $s\overset{n}{\underset{N}{⇄}}d$, where *N* is the set of nodes between source node *s* and node *n*. The soundness of the operator is claimed through the closed-form Theorem.

**Theorem 1.**  *Having $\Xi \equiv \{\Phi \cup \Omega \cup \Psi \cup \Psi^{T}\}$, N×N∈Ξ and 1≤n≤∥C∥+∥E∥$s\overset{n}{\underset{N}{⇄}}d)$ is sound if and only if:*

*1.* 
∀s,d∈(C∪E),∃a∈(C∪E)∥a∈[s〉andd∈[a〉

*2.* 
∀s,d∈(C∪E),∃a,b∈(C∪E)∥a∈[s〉andd∈[b〉andb∈[a•〉anda∈[b•〉

The first condition of the theory ensures a valid connectivity and the second one ensures the nonexistence of cycles in the graph. We will start proving that if the operator is sound then the conditions apply.

**Proof.**  $∵(\overset{k}{\underset{\Xi }{\Leftrightarrow }}\Xi )$ is sound,

∴∃s∈Ξ,d∈Ξ∥d∈[s〉

∵d∈[s〉

$∴\exists a^{*}\in \Xi ∥a^{*}\in [s\rangle \;\mathrm{and}\;d\in [a^{*}\rangle$

$∵(\overset{k}{\underset{\Xi }{\Leftrightarrow }}\Xi )$

$∴(\overset{k}{\underset{\Xi }{\Leftrightarrow }}\Xi )$ will eventually terminate.

∴∀p∈P,p is not cyclic

∴∀a,b∈p,p∈P,ifb∈[a•〉thena∉[b•〉    □

Now, we will prove that if all conditions apply then the operator is sound.

**Proof.** 
∵∀s,d∈(C∪E),∃a∈(C∪E)∥a∈[s〉andd∈[a〉

∴d∈[s〉

∵∀s,d∈(C∪E),∃a,b∈(C∪E)∥a∈[s〉andd∈[b〉andb∈[a•〉anda∈[b•〉

∴∀p∈P,p is acyclic.

$∴(\overset{k}{\underset{\Xi }{\Leftrightarrow }}\Xi )$ is sound    □

### 5.4. Example of Using the Topology Operator

**Figure 3:**
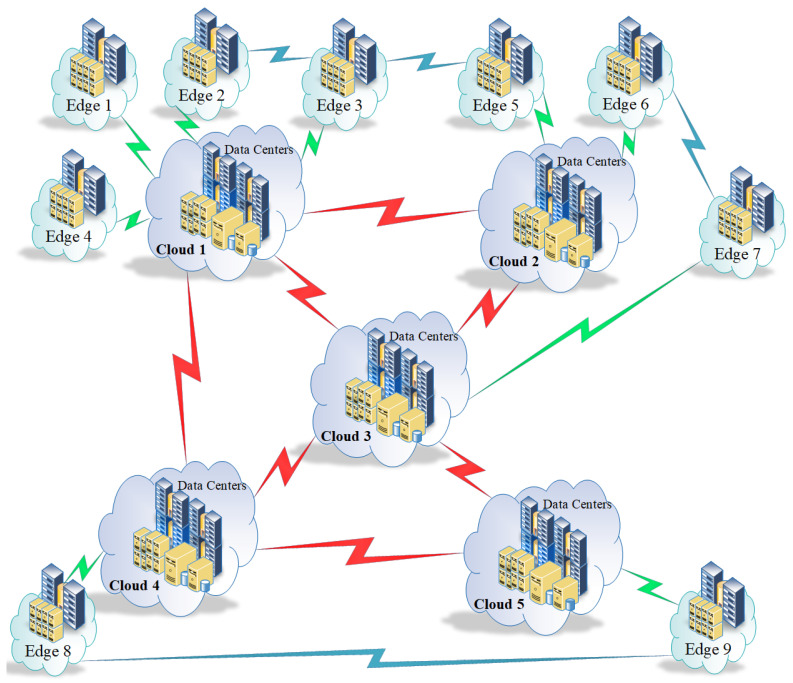
A system Topology.

In this section, an example is given about how operator is used. It is applied on the topology shown in Figure 3 We start with phase 1: Φ paths with 1st degree

(23) $$c_{1}\overset{1}{\underset{\Phi }{⇄}}c_{2}=\Phi \left[1\right]\;\wedge \;\Phi \left[2\right]=\left[\;1\;1\;0\;0\;0\;\right]$$

The result of calculating $c_{1}\overset{1}{\underset{\Phi }{⇄}}c_{2}$ in Equation (23) shows that $c_{1}$ and $c_{2}$ are directly connected but there is no third node that immediately connects them together.

(24) $$c_{1}\overset{1}{\underset{\Phi }{⇄}}c_{3}=\Phi \left[1\right]\;\wedge \;\Phi \left[3\right]=\left[\;1\;0\;1\;0\;0\;\right]$$

The result of calculating $c_{1}\overset{1}{\underset{\Phi }{⇄}}c_{3}$ in Equation (24) shows that $c_{1}$ and $c_{3}$ are directly connected but there is no third node that immediately connects them together.

(25) $$c_{1}\overset{1}{\underset{\Phi }{⇄}}c_{4}=\Phi \left[1\right]\;\wedge \;\Phi \left[4\right]=\left[\;0\;1\;1\;0\;0\;\right]$$

The result of calculating $c_{1}\overset{1}{\underset{\Phi }{⇄}}c_{4}$ in Equation (25) shows that $c_{1}$ and $c_{4}$ are not directly connected. However, they are connected through the 1st degree set $\left\{c_{2},c_{3}\right\}$, which means $c_{1}$ and $c_{4}$ are reachable from each other either through intermediate cloud $c_{2}$ or through intermediate node $c_{3}$.

(26) $$c_{1}\overset{1}{\underset{\Phi }{⇄}}c_{5}=\Phi \left[1\right]\;\wedge \;\Phi \left[5\right]=\left[\;0\;0\;0\;0\;0\;\right]$$

The result of calculating $c_{1}\overset{1}{\underset{\Phi }{⇄}}c_{5}$ in Equation (26) shows that $c_{1}$ and $c_{5}$ are not directly connected and there is no 1st degree connection that binds them together. We assume that connectivity is bi-directional, in other words, if $c_{i}$ is reachable from $c_{j}$ then $c_{j}$ is reachable from $c_{i}$. Based on this assumption, there is no need to test a node $c_{i}$ with any node that has a lower index and that is why for $c_{2}$ we start testing from $c_{3}$.

(27) $$c_{2}\overset{1}{\underset{\Phi }{⇄}}c_{3}=\Phi \left[2\right]\;\wedge \;\Phi \left[3\right]=\left[\;1\;0\;0\;1\;0\;\right]$$

The result of calculating $c_{2}\overset{1}{\underset{\Phi }{⇄}}c_{3}$ in Equation (27) shows that $c_{2}$ and $c_{4}$ are not directly connected but there are intermediate nodes that connect them together, namely $c_{1}$ and $c_{4}$.

(28) $$c_{2}\overset{1}{\underset{\Phi }{⇄}}c_{4}=\Phi \left[2\right]\;\wedge \;\Phi \left[4\right]=\left[\;0\;1\;0\;1\;0\;\right]$$

The result of calculating $c_{2}\overset{1}{\underset{\Phi }{⇄}}c_{4}$ in Equation (28) shows that $c_{2}$ and $c_{4}$ are directly connected and there are no intermediate nodes that bind them together.

(29) $$c_{2}\overset{1}{\underset{\Phi }{⇄}}c_{5}=\Phi \left[2\right]\;\wedge \;\Phi \left[5\right]=\left[\;0\;0\;0\;1\;0\;\right]$$

The result of calculating $c_{2}\overset{1}{\underset{\Phi }{⇄}}c_{5}$ in Equation (29) shows that $c_{2}$ and $c_{5}$ are not directly connected but they are connected through intermediate node $c_{4}$.

(30) $$c_{3}\overset{1}{\underset{\Phi }{⇄}}c_{4}=\Phi \left[3\right]\;\wedge \;\Phi \left[4\right]=\left[\;0\;0\;1\;1\;0\;\right]$$

The result of calculating $c_{3}\overset{1}{\underset{\Phi }{⇄}}c_{4}$ in Equation (30) shows that $c_{3}$ and $c_{4}$ are directly connected and there are no intermediate nodes that indirectly link them together.

(31) $$c_{3}\overset{1}{\underset{\Phi }{⇄}}c_{5}=\Phi \left[3\right]\;\wedge \;\Phi \left[5\right]=\left[\;0\;0\;0\;1\;0\;\right]$$

The result of calculating $c_{3}\overset{1}{\underset{\Phi }{⇄}}c_{5}$ in Equation (31) shows that $c_{3}$ and $c_{5}$ are not directly connected but they are indirectly connected through intermediate node $c_{4}$.

(32) $$c_{4}\overset{1}{\underset{\Phi }{⇄}}c_{5}=\Phi \left[4\right]\;\wedge \;\Phi \left[5\right]=\left[\;0\;0\;0\;1\;1\;\right]$$

Now we continue with phase 2: Φ paths with 2nd degree. In this phase, only those connected nodes in first phase are used. In other words, the path segment between $c_{1}$ and $c_{5}$ in Equation (26) will be ignored since there is no 1st degree connectivity between both nodes.

Applying 2nd degree of the above operation works as follows:

(33) $$\left(c_{i}\overset{n}{\underset{\Xi }{⇄}}c_{j}\right)=\left(c_{i}\overset{n-1}{\underset{\Xi }{⇄}}c_{k}\right)\wedge c_{j}$$

Applying operator $\overset{n-1}{\underset{\Xi }{⇄}}$ in phase 2 gives one of three different possible outputs:

1. A vector that contains zeros
2. A vector that contains a value one that represents the source and every other value is zero
3. otherwise

Case 1 means that source and destination are completely disconnected through the path tested by source $\overset{n-1}{\underset{\Xi }{⇄}}$ destination. Case 2 means that source and destination are directly connected and there is no other connection path. Case 3 is the only case that will propagate to further phases. The generation of 2nd degree paths is as follows:

(34) $$\left(c_{1}\overset{2}{\underset{\Phi }{⇄}}c_{2}\right)=\left\{\begin{array}{c} \left(c_{1}\overset{1}{\underset{\Phi }{⇄}}c_{3}\right)\wedge c_{2}\\ \left(c_{1}\overset{1}{\underset{\Phi }{⇄}}c_{4}\right)\wedge c_{2} \end{array}\right.$$

(35) $$\left(c_{1}\overset{1}{\underset{\Phi }{⇄}}c_{3}\right)\wedge c_{2}=\left[\;1\;0\;1\;0\;0\;\right]\wedge \left[\;1\;1\;0\;1\;0\;\right]=\left[\;1\;0\;0\;0\;0\;\right]$$

The result tells that path $\left(c_{1}\overset{c_{3}}{\underset{\Phi }{\Leftrightarrow }}c_{2}\right)$ will not be considered for further phases since it does not add any information. The result shows that there is a direct path between $c_{1}$ and $c_{3}$.

(36) $$\left(c_{1}\overset{1}{\underset{\Phi }{⇄}}c_{4}\right)\wedge c_{2}=\left[\;0\;1\;1\;0\;0\;\right]\wedge \left[\;1\;1\;0\;1\;0\;\right]=\left[\;0\;1\;0\;0\;0\;\right]$$

Path $\left(c_{1}\overset{c_{4}}{\underset{\Phi }{\Leftrightarrow }}c_{2}\right)$ will be used in the 3rd phase since it adds a path to $c_{2}$ through $c_{4}$.

Note that $\left(c_{1}\overset{1}{\underset{\Phi }{⇄}}c_{5}\right)$ is ignored since it is a 0. The 2nd degree path to $c_{3}$ from $c_{1}$ is found as follows:

(37) $$\left(c_{1}\overset{2}{\underset{\Phi }{⇄}}c_{3}\right)=\left\{\begin{array}{c} \left(c_{1}\overset{1}{\underset{\Phi }{⇄}}c_{2}\right)\wedge c_{3}\\ \left(c_{1}\overset{1}{\underset{\Phi }{⇄}}c_{4}\right)\wedge c_{3} \end{array}\right.$$

(38) $$\left(c_{1}\overset{1}{\underset{\Phi }{⇄}}c_{2}\right)\wedge c_{3}=\left[\;1\;1\;0\;0\;0\;\right]\wedge \left[\;1\;0\;1\;1\;0\;\right]=\left[\;1\;0\;0\;0\;0\;\right]$$

Results indicate that path $\left(c_{1}\overset{c_{2}}{\underset{\Phi }{\Leftrightarrow }}c_{3}\right)$ will not be considered in the 3rd phase since it does not add new information.

(39) $$\left(c_{1}\overset{1}{\underset{\Phi }{⇄}}c_{4}\right)\wedge c_{3}=\left[\;0\;1\;1\;0\;0\;\right]\wedge \left[\;1\;0\;1\;1\;0\;\right]=\left[\;0\;0\;1\;0\;0\;\right]$$

Results indicate that path $\left(c_{1}\overset{c_{4}}{\underset{\Phi }{\Leftrightarrow }}c_{3}\right)$ is used in the 3rd phase since $c_{3}$ is reachable from $\left(c_{1}\overset{1}{\underset{\Phi }{⇄}}c_{4}\right)$ information.

The 2nd degree path to $c_{4}$ from $c_{1}$ is presented as follows:

(40) $$\left(c_{1}\overset{2}{\underset{\Phi }{⇄}}c_{4}\right)=\left\{\begin{array}{c} \left(c_{1}\overset{1}{\underset{\Phi }{⇄}}c_{2}\right)\wedge c_{4}\\ \left(c_{1}\overset{1}{\underset{\Phi }{⇄}}c_{3}\right)\wedge c_{4} \end{array}\right.$$

(41) $$\left(c_{1}\overset{1}{\underset{\Phi }{⇄}}c_{2}\right)\wedge c_{4}=\left[\;1\;1\;0\;0\;0\;\right]\wedge \left[\;0\;1\;1\;1\;1\;\right]=\left[\;0\;1\;0\;0\;0\;\right]$$

Results indicate that $c_{4}$ is reachable which means that path $\left(c_{1}\overset{c_{2}}{\underset{\Phi }{\Leftrightarrow }}c_{4}\right)$ will be considered in the 3rd phase.

(42) $$\left(c_{1}\overset{1}{\underset{\Phi }{⇄}}c_{3}\right)\wedge c_{4}=\left[\;1\;0\;1\;0\;0\;\right]\wedge \left[\;0\;1\;1\;1\;1\;\right]=\left[\;0\;0\;1\;0\;0\;\right]$$

Results indicate that $c_{4}$ is reachable from $\left(c_{1}\overset{1}{\underset{\Phi }{⇄}}c_{3}\right)$ which means that path $\left(c_{1}\overset{c_{3}}{\underset{\Phi }{\Leftrightarrow }}c_{4}\right)$ will be considered in the 3rd phase.

The 2nd degree path to $c_{5}$ from $c_{1}$ is found as follows:

(43) $$\left(c_{1}\overset{2}{\underset{\Phi }{⇄}}c_{5}\right)=\left\{\begin{array}{c} \left(c_{1}\overset{1}{\underset{\Phi }{⇄}}c_{2}\right)\wedge c_{5}\\ \left(c_{1}\overset{1}{\underset{\Phi }{⇄}}c_{3}\right)\wedge c_{5}\\ \left(c_{1}\overset{1}{\underset{\Phi }{⇄}}c_{4}\right)\wedge c_{5} \end{array}\right.$$

(44) $$\left(c_{1}\overset{1}{\underset{\Phi }{⇄}}c_{2}\right)\wedge c_{5}=\left[\;1\;1\;0\;0\;0\;\right]\wedge \left[\;0\;0\;0\;1\;1\;\right]=\left[\;0\;0\;0\;0\;0\;\right]$$

This means that path $\left(c_{1}\overset{c_{2}}{\underset{\Phi }{\Leftrightarrow }}c_{5}\right)$ will not be used in the 3rd phase.

(45) $$\left(c_{1}\overset{1}{\underset{\Phi }{⇄}}c_{3}\right)\wedge c_{5}=\left[\;1\;0\;1\;0\;0\;\right]\wedge \left[\;0\;0\;0\;1\;1\;\right]=\left[\;0\;0\;0\;0\;0\;\right]$$

This means that path $\left(c_{1}\overset{c_{3}}{\underset{\Phi }{\Leftrightarrow }}c_{5}\right)$ will not be used in the 3rd phase.

(46) $$\left(c_{1}\overset{1}{\underset{\Phi }{⇄}}c_{4}\right)\wedge c_{5}=\left[\;0\;1\;1\;0\;0\;\right]\wedge \left[\;0\;0\;0\;1\;1\;\right]=\left[\;0\;0\;0\;0\;0\;\right]$$

This means that path $\left(c_{1}\overset{c_{4}}{\underset{\Phi }{\Leftrightarrow }}c_{5}\right)$ will not be used in the 3rd phase. This will be repeated for paths that start with $c_{2},c_{3},c_{4}\;\mathrm{and}\;c_{5}$. Only successful paths will continue for phase 3. Note that the maximum number of phases is *n*, where *n* is the number of clouds connected to the topology Φ. The stopping criterion of the algorithm is the failure to produce any vector that can continue to the next phase.

#### 5.4.1. Sub-Topologies

The previous demonstration for Φ applies perfectly to Ω since both Φ and Ω are both square matrices. Ψ represents the connectivity between clouds and edge servers which means that it is not necessarily a square matrix. If we want to find the connectivity between two clouds through one or more intermediate edge servers the algorithm still applies the same. However, if we need to find the connectivity between two edge servers through two or more clouds we need to use $\Psi^{T}$.

#### 5.4.2. Over All Algorithm

The algorithm finds paths that include diversity of different types of nodes. In other words, the path is a mix between links that bind clouds, edge servers, or clouds and edge servers together. The formula for the algorithm is defined to be:

(47) $$\forall p\in P,p\in \Xi {\left(\left(\overset{k}{\underset{\Xi }{\Leftrightarrow }}\Xi \right){\left(\Phi^{*}\cup \Omega^{*}\cup \Psi^{*}\cup {\left(\Psi^{T}\right)}^{*}\right)}^{+}\right)}^{*}$$

The equation means that there are paths such that every path has a minimum of one connection that could be:

1. Connection from a cloud to a cloud
2. Connection from an edge server to an edge
3. Connection from a cloud to edge server
4. Connection from edge server to a cloud

Mathematically, let us assume that we have a path from cloud $c_{1}$ to cloud $c_{2}$, then to edge $e_{1}$, then to edge server $e_{2}$ and finally to cloud $c_{4}$. This will be represented as follows:

(48) $$\left(\left(\Phi \left[1\right]\wedge \Phi \left[2\right]\right)\wedge \Psi^{T}\left[1\right])\wedge \Omega \left[2\right]\wedge \Phi \left[4\right]\right)$$

While matrices here have different dimensionality, what matters here is the dimensional equality of every two consecutive matrices since every operation verifies whether there is a path or not.

Note that the simplest way is to directly use Ξ in the format given in Equation (15) shown in Equations (21) and (22) because it is a square matrix and the application of the algorithm and the operator becomes very straightforward without the need to use Equation (47) which deal with heterogeneous non-uniform matrix cases.

Algorithm 1 takes incident matrix Ξ, the source node *s* and the destination node *d* as inputs and returns all possible paths *s* to *d*. Note that every path has the information of transmission cost in seconds between every two successive nodes per bit. This means that the longer the data to be transmitted are, the higher the cost is. The transmission cost differs from node to node and the total cost when transmitting *b* bits from source *s* to destination *d* through path *p* is presented as follows:

(49) $$cost=\Sigma \tau \left(c_{i},c_{j}\right)\times b∥\left(c_{i},c_{j}\right)\in p,\;\mathrm{and}\;c_{j}=c_{i}•,s=c_{i}∥i=0\;\mathrm{and}\;d=c_{j}∥j=∥p∥$$

where $c_{i}•$ is the successor of $c_{i}$. After acquiring the different paths, we pick the path with the shortest cost, and note that the cost here is not just transmission cost, it is also the replication cost of replication cost. The minimum cost now is presented as follows:

(50) $$min\left(cost\right)=min(\tau \left(c_{s},c_{j}\right)+\pi \left(c_{j}\right)+\rho \left(c_{j}\right)+\Sigma_{i=1}^{t}\tau \left(c_{s},c_{j}\right))$$

where *t* is the number of times node $c_{d}$ will communicate with node $c_{j}$.

The proposed algorithm is an algorithm that finds the cost of different paths from one node to another. The algorithm is divided into two phases: The topology recognition phase and the dynamic behaviour phase. The topology phase literally builds the graph while the dynamic behaviour phase keeps watching costs and finds the minimum path accordingly. When comparing this algorithm to the traditional Dijkstra graph minimisation [52]. We can see the following:

1. The objective of Dijkstra is learning the path with the minimum cost and the cost is considered static and if cost changes, the algorithm is being applied again from the starting point until reaching the destination. The objective of the proposed algorithm is to learn the topology and then assign dynamic costs that varies from one point of time to another and according to changes that occurs, file segments begin to be replicated or deleted from certain nodes.
2. While the Dijkstra algorithm works on undirected graphs, it is not guaranteed to be sound when weights are negative. In the proposed algorithm, this cannot happen since nodes that have been visited before cannot be visited again. This is guaranteed by the algorithm itself and verified by theory Section 5.3.
3. The proposed algorithm learns the topology and then when costs change, only those nodes that are effected with be notified and therefore the service requester will move to another path. This also means that replicating a file segment in a node or removing a segment from a node is also handled by the proposed algorithm.

**Algorithm 1** All paths from node *s* to node *d*
**Require:** Incident Matrix Ξ, source *s* and destination *d*  1:**function** find-paths-recursive(Ξ,AllPaths,CurrentPath,s,d)  2:    **let** index←0  3:    **for every** ξ∈Ξ **do**  4:        **let** index←index+1  5:        **let** $path_{t}\leftarrow CurrentPath$  6:        **if** ξ∉path **then**  7:           **if** index(ξ)==index(d) **then**  8:               **let** $path_{t}\leftarrow path_{t}\cup d$  9:               **if** $path_{t}\notin AllPaths$ **then**10:                   **let** $AllPaths\leftarrow AllPaths\cup path_{t}$11:               **end if**12:           **else if** ξ(index)==1 **then**13:               **let** $path_{t}\leftarrow CurrentPath$14:               **let** $path_{t}\leftarrow path_{t}\cup \xi$15:               find-paths-recursive($\Xi ,AllPaths,path_{t},s,d$)16:           **end if**17:        **end if**18:    **end for**19:    **return** AllPaths20:**end function**

## 6. File Replication

Back to Equation (1), the third component of the system is F, the set of files described in Equation (4) which are accessed in the System Ξ. The cost of accessing file f∈F depends on many factors including how far the file from the destination node α∈Ω and whether that α is a cloud or an edge server. In this paper, we assume that the total cost η of file replication is to be the data transfer delay that will be saved in τ, in addition to, the data processing cost π and the load on the hosting node ρ. These factors are weighed and weights differ from a server to another.

(51) $$\eta =\beta_{1}\pi +\beta_{2}\rho +\beta_{3}\tau$$

The unit costs for every independent variable in Equation (51) is determined by the host and the weights are learned through historical data. When replicating a file f∈F, the cost of data transfer and the hosting depend on how big the file is, this means that replicating segments of a file could minimise both hosting and data transfer costs. File is being segmented to a number of segments according to accessibility. For a region *s* of a large file *f*, if this region is frequently accessed, this *S* will be a separate segment which means file will be divided into three segments, segment $s_{b}$ which is the portion before the target segment, segment *s* which is the required one and segment $s_{a}$ which is the part of the file after the required segment. This means that file *f* after partition is:

$$f=\{s_{b},s,s_{a}\}.$$

A segment can be partitioned into several sub segments the same way. The details of how this is carried out is being handled in a future study and in this paper we assume that files are already partitioned.

## 7. Simulation and Results

In our simulation, we apply the proposed framework on the system shown in Figure 3 with the setup in Table 1 and Table 2. We assume that we have three different files $f_{1},f_{2},f_{3}$, where these files are divided into segments as follows:

$f_{1}$ is divided into two segments

$$f_{1}=\left\{s_{11},s_{12}\right\}$$

$f_{2}$ is divided into three segments

$$f_{2}=\left\{s_{21},s_{22},s_{23}\right\}$$

$f_{3}$ is divided into four segments

$$f_{2}=\left\{s_{31},s_{32},s_{33},s_{34}\right\}$$

Table 1 shows the allocation of different file segments in different clouds and edge servers. Segment $s_{11}\in f_{1}$ is located in three different nodes, namely, $c_{1},c_{2}$ and $e_{7}$. File segment $s_{12}\in f_{1}$ is located only in $c_{1}$. File segments $s_{21},s_{22}\in f_{2}$ are located only in $c_{2}$ while file segment $s_{23}\in f_{2}$ is located in both $c_{2}$ and $c_{4}$. File segment $s_{31}\in f_{3}$ is located only in $c_{3}$. File segment $s_{32}\in f_{3}$ is located in both clouds $c_{3}$ and $c_{5}$. Last but not least, file segments $s_{33},s_{34}\in f_{3}$ are both located in cloud $c_{3}$. From the data given in Table 1, it is easy to conclude that $f_{1}$ exists originally in $c_{1}$ since replication does not delete original segments and since $s_{12}$ exists only in $c_{1}$. This means that $s_{11}$ was replicated to both nodes $e_{7}$ and $c_{2}$. The same applies for $f_{2}$. It is obvious that $f_{2}$ was originally located in $c_{2}$ and $s_{23}$ was replicated in $c_{4}$ since the whole file $f_{2}=\left\{s_{21},s_{22},s_{23}\right\}$ exists in $c_{2}$ and $s_{23}$ is the only segment of file $f_{2}$ that exists in $c_{4}$ as seen in Table 1. Investigating $f_{3}$, it is obvious that it originally existed in cloud $c_{3}$ and then $s_{32}$ was replicated to $c_{5}$ since the whole file $f_{3}=\left\{s_{31},s_{32},s_{33},s_{34}\right\}$ exists in $c_{3}$ and $s_{32}$ is the only segment of file $f_{3}$ that exists in $c_{5}$ as seen in Table 1. Table 2 shows the assumed file segment lengths, where file length is calculated as follows:

(52) $$\kappa \left(f_{i}\right)=\Sigma_{j=1}^{n}\kappa \left(s_{ij}\right)$$

This means that $\kappa \left(f_{1}\right)=\kappa \left(s_{11}\right)+\kappa \left(s_{12}\right)=100+150=250$, $\kappa \left(f_{2}\right)=\kappa \left(s_{21}\right)+\kappa \left(s_{22}\right)+\kappa \left(s_{23}\right)=60+110+210=360$ and $\kappa \left(f_{3}\right)=\kappa \left(s_{31}\right)+\kappa \left(s_{32}\right)+\kappa \left(s_{33}\right)+\kappa \left(s_{34}\right)=50+20+200+120=390$ where κ is the length in bits.

Matrix τ in Equation (53) shows the assumed communication delays in milliseconds. τ is a square matrix that defines the communication delays between every two nodes. If the two nodes are not directly connected, the cost is *∞* since it is a topology matrix and it recognises only the connectivity between different nodes whereas if it is directly connected, the delay is considered. Note that the diagonal of the matrix is 0 since there is no delay for any node to itself.

(53) $$\tau =\left[\begin{array}{cccccccccccccc} 0 & 6 & 5 & 3 & \infty & 13 & 12 & 10 & 14 & \infty & \infty & \infty & \infty & \infty \\ 6 & 0 & 6 & \infty & \infty & \infty & \infty & \infty & \infty & 16 & 13 & \infty & \infty & \infty \\ 5 & 6 & 0 & 7 & 8 & \infty & \infty & \infty & \infty & \infty & \infty & 14 & \infty & \infty \\ 3 & \infty & 7 & 0 & 8 & \infty & \infty & \infty & \infty & \infty & \infty & \infty & 17 & \infty \\ \infty & \infty & 8 & 8 & 0 & \infty & \infty & \infty & \infty & \infty & \infty & \infty & \infty & 12\\ 13 & \infty & \infty & \infty & \infty & 0 & \infty & \infty & \infty & \infty & \infty & \infty & \infty & \infty \\ 12 & \infty & \infty & \infty & \infty & \infty & 0 & 18 & \infty & \infty & \infty & \infty & \infty & \infty \\ 10 & \infty & \infty & \infty & \infty & \infty & 18 & 0 & \infty & 20 & \infty & \infty & \infty & \infty \\ 14 & \infty & \infty & \infty & \infty & \infty & \infty & \infty & 0 & \infty & \infty & \infty & \infty & \infty \\ \infty & 16 & \infty & \infty & \infty & \infty & \infty & 20 & \infty & 0 & \infty & \infty & \infty & \infty \\ \infty & 13 & \infty & \infty & \infty & \infty & \infty & \infty & \infty & \infty & 0 & 15 & \infty & \infty \\ \infty & \infty & 14 & \infty & \infty & \infty & \infty & \infty & \infty & \infty & 15 & 0 & \infty & \infty \\ \infty & \infty & \infty & 17 & \infty & \infty & \infty & \infty & \infty & \infty & \infty & \infty & 0 & 19\\ \infty & \infty & \infty & \infty & 12 & \infty & \infty & \infty & \infty & \infty & \infty & \infty & 19 & 0 \end{array}\right]$$

For $e_{9}$ to access $s_{11}$, we have three different paths:

1. $p_{1}\equiv c_{1}\to c_{4}\to c_{5}\to e_{9}$
2. $p_{2}\equiv c_{2}\to c_{3}\to c_{5}\to e_{9}$
3. $p_{3}\equiv e_{7}\to c_{3}\to c_{5}\to e_{9}$

The cost for $e_{9}$ to access $s_{11}$*n* times through path $p_{1}$ when hosting in an intermediate node $c_{5}$ is as follows:

$$cost=\tau \left(c_{1},c_{5}\right)+\rho \left(c_{5}\right)+\pi \left(c_{5}\right)+n\times \tau \left(c_{5},e_{9}\right)$$

**Figure 4:**
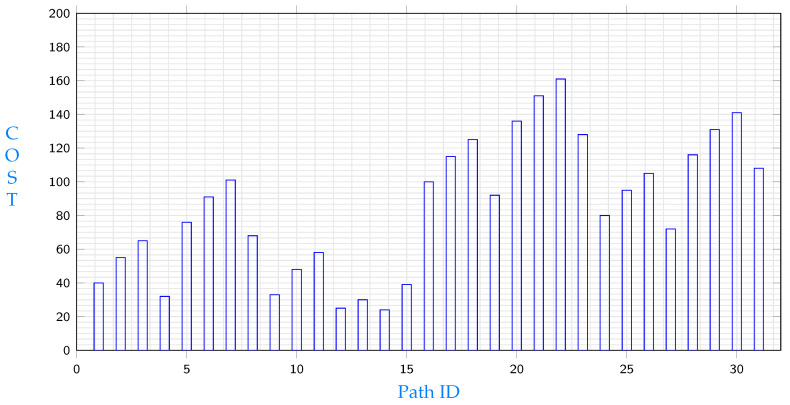
Path costs from $c_{1}$ to $e_{9}$.

The following experiment shows the results of node $e_{9}$ requesting file segment $s_{11}$. This file segment exists in three different locations according to Table 1. Those locations are $c_{1},c_{2}$ and $e_{7}$ and paths for each source are studied below. The costs of different paths from $c_{1}$ to $e_{9}$ are shown in Figure 4. Among paths that start with node $c_{1}$, the path with the minimum cost is:

$$c_{1}\to c_{4}\to c_{5}\to e_{9}$$

Figure 4 shows the lowest cost paths from $c_{1}$ to $e_{9}$. It is obvious that total costs differ from a path to another. Note that costs in the same path could differ depending on which node will be selected for replication.

**Figure 5:**
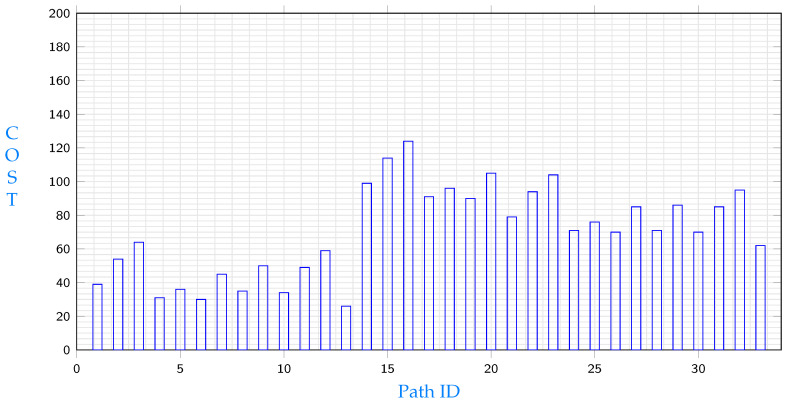
Path costs from $c_{2}$ to $e_{9}$.

Costs of different paths from $c_{2}$ to $e_{9}$ is shown in Figure 5. Among paths that start with node $c_{2}$, the path with the minimum cost is:

$$c_{2}\to c_{3}\to c_{5}\to e_{9}$$

**Figure 6:**
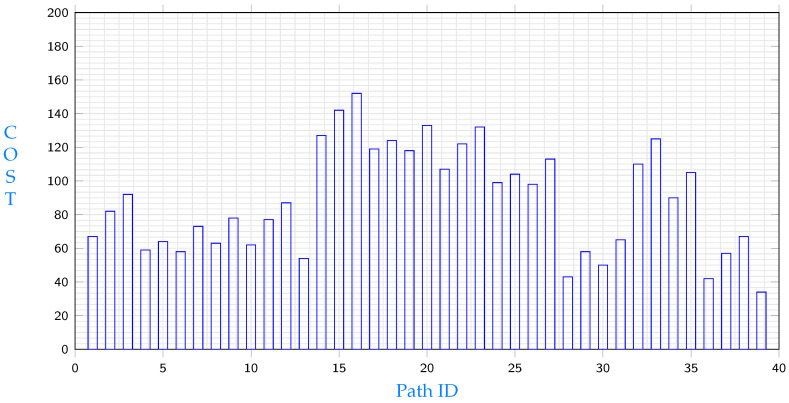
Path costs from $e_{7}$ to $e_{9}$.

Figure 5 shows the lowest cost paths from $c_{2}$ to $e_{9}$. Again, it is obvious that total costs differ from a path to another. Costs in the same path could differ depending on which node will be selected for replication. Costs of different paths from $e_{7}$ to $e_{9}$ is shown in Figure 6. Among paths that start with node $e_{7}$, the path with the minimum cost is:

$$e_{7}\to c_{3}\to c_{5}\to e_{9}$$

**Figure 7:**
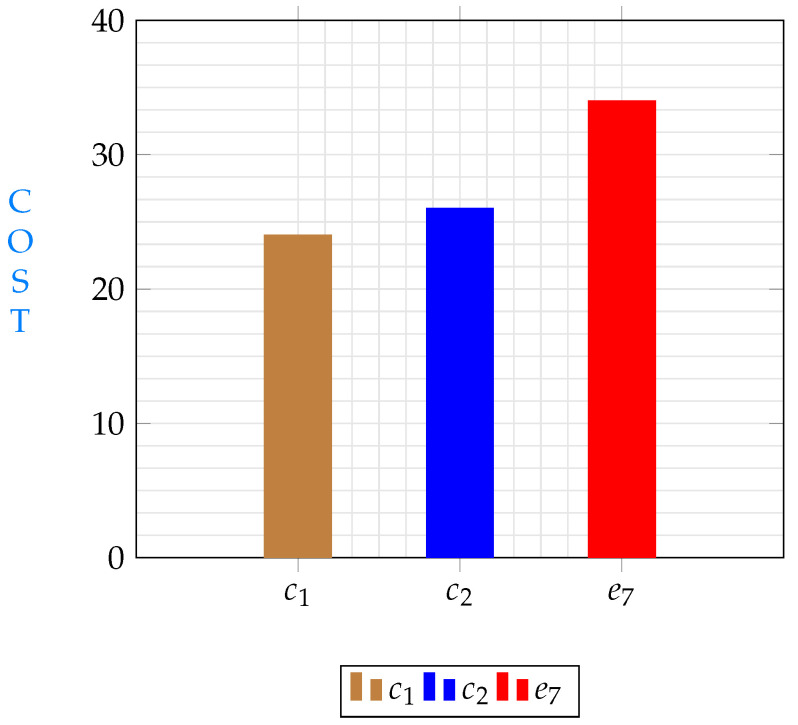
Minimum costs when streaming from different nodes.

Figure 6 shows the lowest cost paths from $e_{7}$ to $e_{9}$. Again, it is obvious that total costs differ from a path to another. Costs in the same path could differ depending on which node will be selected for replication. Data in Figure 4, Figure 5 and Figure 6 illustrate different path costs from source to destination. Every bar represents total cost of a path. Those costs are calculated using Equation (51). Lengths of segments are shown in Table 2, communication delays are shown in Equation (53) and the hosting cost is being calculated based on the segment hosting shown in Table 1. A comparison with the three minimums resulting from different starting points shows different costs for the three shortest paths are shown in Figure 7, the comparison shows that replicating $s_{11}$ from node $c_{1}$ will give the minimum cost.

Now we show the selection of the best candidate to host the replica of $s_{11}$ requested by $e_{9}$. Sources without replication are $c_{1},c_{2}$ and $e_{7}$.

Now that we know the chosen path is:

$$c_{1}\to c_{4}\to c_{5}\to e_{9}$$

**Figure 8:**
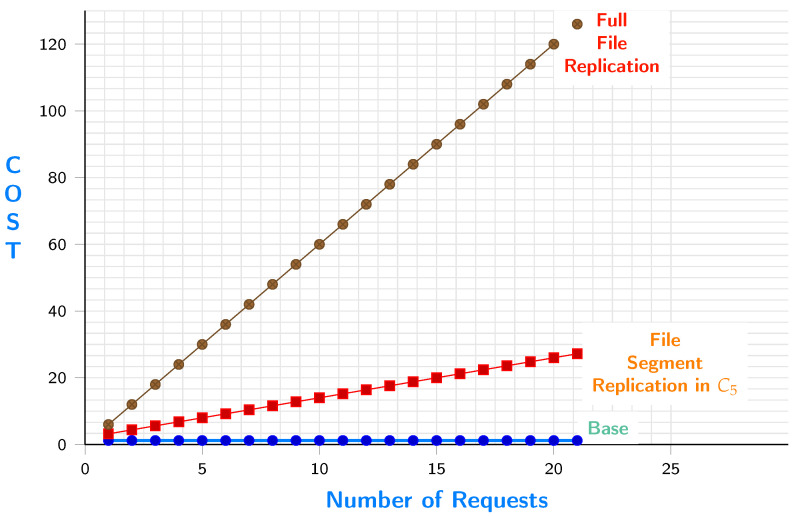
File segment requests when replicated in $c_{5}$ VS. all file requests.

The replication could occur to $c_{4}$ or $c_{5}$. Figure 8 shows the cost of replicating $s_{11}$ to $c_{5}$ versus replicating the whole file. The figure shows the base cost, which is the cost of moving data from source $c_{1}$ to $c_{5}$. It is low because it happens only once. The segment replication increases with a low slope since we only replicate what is requested by $e_{9}$. The third line has a high slope since the whole file is being accessed every time $e_{9}$ sends a request.

**Figure 9:**
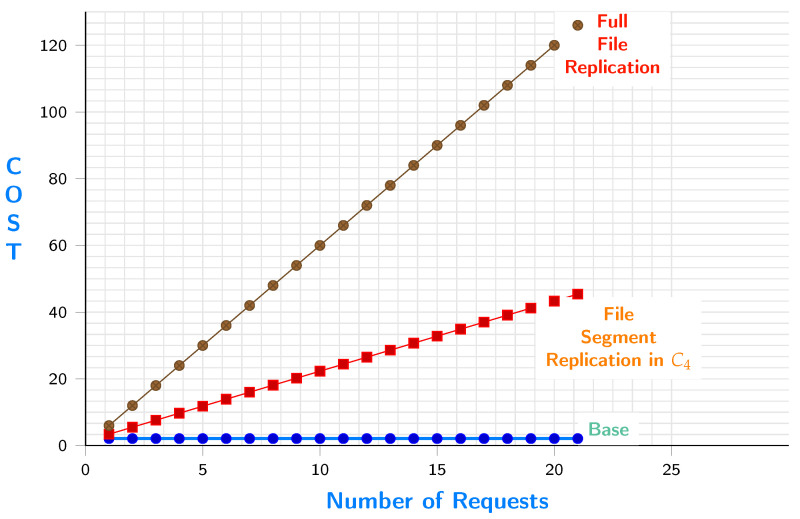
File segment requests when replicated in $c_{4}$ VS. all file requests.

Figure 9 shows the cost of replicating $s_{11}$ to $c_{4}$ versus replicating the whole file. The figure shows the base cost which is the cost of moving data from source $c_{1}$ to $c_{4}$. It is low because it happens only once, and the segment replication increases with a low slope since we only replicate what is needed by $e_{9}$. The third line has a high slope since the whole file is being accessed every time $e_{9}$ sends a request.

**Figure 10:**
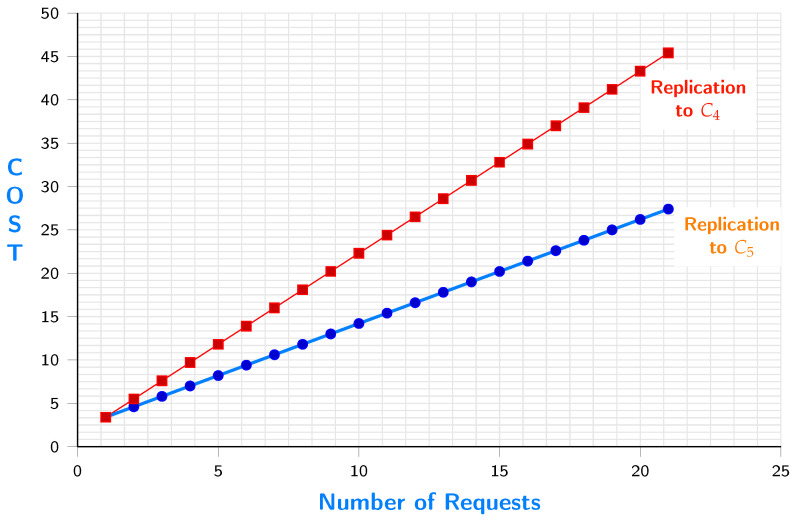
Requestcost when replicating to $c_{5}$ vs replicating to $c_{4}$.

Figure 10 shows the difference of replication cost between $c_{4}$ and $c_{5}$. The results show that the more the iterations are, the lower the cost is when replicating to $c_{5}$.

This study employs a multivariate regression model as a machine learning technique to identify the optimal replica. The approach involves calculating the cost associated with each node if it were to run the process and factoring in the total cost of replication, taking into account the size of the file’s segments to be replicated. By utilising this approach, we aim to determine the optimal choice of a replica in a cost-effective manner. As a sample of the data, we provide a subset of the training data:

Table 3 showcases a small subset of the training data employed in this study. The total number of rows in the dataset was 10,000, which is considered sufficient to achieve a mean square error below our predefined minimum threshold. The first column of the table represents the processor used for a given task. The discrete values in this column represent three different types of processors, with 1 indicating the slowest and 3 indicating the fastest. The second column displays the $log_{2}$ of the memory size in gigabytes for the task. The third column shows the percentage of memory used for the given task. The value of the used memory is displayed in $log_{2}$ of the memory used in gigabytes.

The fourth column represents the size of the task required to execute, where the numbers represent the $log_{2}$ of the task length in gigabytes. Note that in this context, the term “task” refers to the entire activity required to execute and it is not related to the definition used in operating systems engineering. Furthermore, the fifth column shows the $log_{2}$ of the disk size in gigabytes, while the sixth column displays the percentage of disk space used for the given task. The value of the used disk space is also displayed in $log_{2}$ of the disk space used in gigabytes.

The seventh column of the table represents the $log_{2}$ of the segment size in gigabytes. Finally, the last column represents the duration in microseconds required to process the given task that accesses the given file segment. It is important to note that this is only a small sample of the complete training dataset, which was utilised to train a multivariate regression model. This model was used to determine the optimal choice of a replica, based on the cost associated with each node if it were to run the process and the total cost of replication. The outcome of the training process is summarised as follows:

Table 4 shows the outcome of the training process. The model with the learned coefficients can be presented as follows:

(54) $$Y=-0.32X_{1}-0.05X_{2}+0.054X_{3}+0.1X_{4}-0.2X_{5}+0.2X_{6}+0.4X_{7}$$

It is noteworthy that the first independent variable in Table 3 is denoted as $X_{1}$, which corresponds to the Processor column. The remaining independent variables continue in the same sequence until column $X_{7}$, which represents the “segment size” variable. Furthermore, the dependent variable “duration” is denoted by Y and is located in the last column of Table 3. Once the time is predicted, the process will need to be executed in certain node, the total cost is predicted to be communication cost and processing cost. Communication cost is already known as mentioned before and processing cost is calculated using Equation (54). The node with the lowest cost is the node that will be chosen for replication and task execution. The model built by machine learning helps choosing the optimal candidate for file segment replication based on history data. The cost that is calculated by regression is the presented by π in Equation (51).

### Performance Comparison between Using ML and Picking a Random Eerver

**Figure 11:**
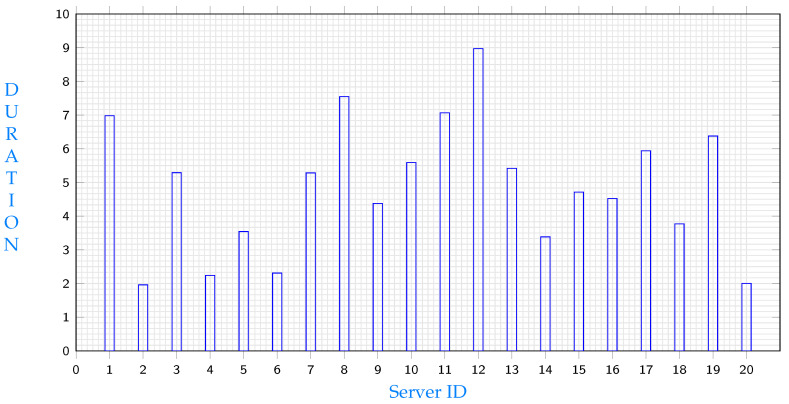
Delays when using different servers.

In this subsection, a comparison is conducted between the performance of the system when utilising machine learning and when selecting a server randomly. Table 5 presents various configurations to choose from, with values that are interpreted similarly to the explanation provided above. The experiment focuses on a process that has a burst of 18 and uses a segment with a size of 5. To predict the duration required to execute a task with these specifications, Equation (54) is utilised. The resulting predicted costs of each server are demonstrated in Figure 11.

As depicted in Figure 11, server 2 offers the shortest duration to execute a task with a burst of 18 and access a file segment of 5. Without utilising machine learning, a random selection of servers would have been made, possibly resulting in a server such as server 12 being chosen. However, executing the same task on server 12 would take almost 4.5 times the duration required on server 2.

**Figure 12:**
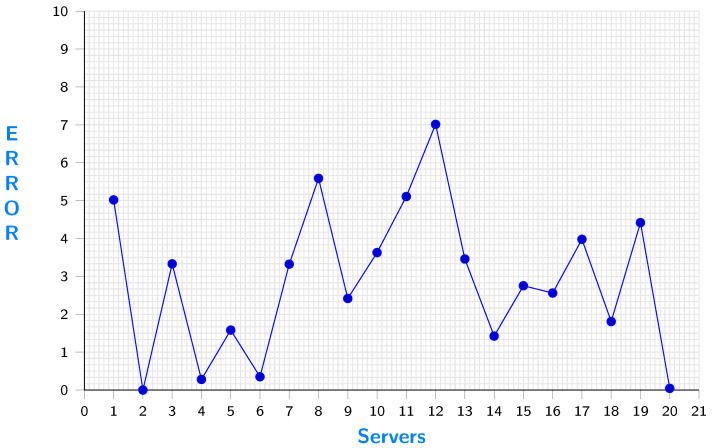
Error based on server.

The error in server selection is demonstrated in Figure 12. In this context, the error of server *s* is defined as the difference between the duration required to execute a task on server *s* and the minimum possible duration achievable with the available servers. As server 2 provides the shortest duration to execute the given task, its error is zero.

## 8. Conclusions

This paper proposes a machine learning approach for optimising file replication in a multi-cloud system in a smart city. Edge servers often communicate with clouds to request data in IoT and smart cities in general. A large amount of data transfer occurs by smart sensors and devices as they request significant amounts of data from different clouds in order to use them in tasks such as interpolation, prediction and decision making. This paper defines a framework for multi-cloud and edge server–cloud collaboration in which they build a virtual workflow to share files. In this paper, the file is divided into segments and segments can be replicated. In other words, there is no need to replicate the whole file if only certain segments are requested in some geographical areas while others are not. In this paper, a mathematical operator is proposed. This operator is applied on a predefined topology. Based on this topology and by applying the operator the needed number of times, the minimum route between some source and destination is found. A closed-form theorem is proposed and proved. The outcome of applying this operator is a cyclic sound path which leads from source to destination. The proposed algorithm finds all the nodes (sources) whether they are clouds or edge servers that host the requested segment. For every source, paths are found and the cost of those paths are calculated. The path is a series of nodes connected together in an acyclic series as mentioned earlier. A node in a path is internal if it belongs to the path and if it is between the source and destination. Every internal node is evaluated for being a hosting candidate for the requested segment based on experience (node history) and the node replies with either accept, neutral or reject. The node that has the minimum total cost from the accepting nodes is selected. If there are no accepting nodes then the one that will guarantee the lowest cost among the neutrals is selected. If there are no neutral nodes then the system will not replicate and the requesting node will stream from one of the sources. This will still guarantee a better performance than full replication if the performance of full replication is measured the same way the algorithm measures the costs. Simulation shows the improvement of system performance when applying the proposed model as compared to full replication. The worst case scenario cost of optimising any selected path by replicating to a node that belong to this path is equivalent to the best case scenario cost of no replication and definitely better than the worst case scenario of replicating the whole file.

## Figures and Tables

**Table 1:** Segment allocation in clouds and edge servers.

	$c_{1}$	$c_{2}$	$c_{3}$	$c_{4}$	$c_{5}$	$e_{1}$	$e_{2}$	$e_{3}$	$e_{4}$	$e_{5}$	$e_{6}$	$e_{7}$	$e_{8}$	$e_{9}$
$s_{11}$	✓	✓	✗	✗	✗	✗	✗	✗	✗	✗	✗	✓	✗	✗
$s_{12}$	✓	✗	✗	✗	✗	✗	✗	✗	✗	✗	✗	✗	✗	✗
$s_{21}$	✗	✓	✗	✗	✗	✗	✗	✗	✗	✗	✗	✗	✗	✗
$s_{22}$	✗	✓	✗	✗	✗	✗	✗	✗	✗	✗	✗	✗	✗	✗
$s_{23}$	✗	✓	✗	✓	✗	✗	✗	✗	✗	✗	✗	✗	✗	✗
$s_{31}$	✗	✗	✓	✗	✗	✗	✗	✗	✗	✗	✗	✗	✗	✗
$s_{32}$	✗	✗	✓	✗	✓	✗	✗	✗	✗	✗	✗	✗	✗	✗
$s_{33}$	✗	✗	✓	✗	✗	✗	✗	✗	✗	✗	✗	✗	✗	✗
$s_{34}$	✗	✗	✓	✗	✗	✗	✗	✗	✗	✗	✗	✗	✗	✗

**Table 2:** File Segment Lengths.

$s_{11}$	$s_{12}$	$s_{21}$	$s_{22}$	$s_{23}$	$s_{31}$	$s_{32}$	$s_{33}$	$s_{34}$
100	150	60	110	210	50	20	200	120

**Table 3:** A small sample of the training data.

Processor	Memory Size	Memory Usage	Task Size	Disk Size	Disk Usage	Segment Size	Duration
2	21	1	15	38	31	1	3.43
2	27	4	18	40	28	9	9.57
3	28	9	9	32	2	16	6.61
1	21	2	18	33	1	21	9.46
1	29	20	1	31	19	6	6.22
2	30	20	9	34	9	20	10.3
3	23	19	2	34	12	16	8.61
3	22	11	1	35	2	19	6.27
2	21	16	4	39	25	3	4.24
1	23	4	15	31	11	13	9.08
1	24	15	2	31	10	11	6.68
3	21	19	1	40	34	3	6.33
2	26	11	2	31	6	7	3.57
1	21	3	3	40	3	18	6.2
1	21	12	7	35	20	11	9.89
3	29	26	1	40	11	11	4.79
1	29	18	6	31	9	9	6.18

**Table 4:** Training process outcome.

Criteria	Value
Training set Length in records	37,325
Testing set Length in records	37,000
intercept	7.24
mean squared error	0.98
mean absolute error	0.62
r2 score	0.94
explained variance	0.94
maximum error	2.12
mean absolute percentage error	0.07

**Table 5:** Different Server configurations.

Server	Processor	Memory Size	Memory Usage	Disk Size	Disk Usage
1	1	23	12	34	24
2	2	28	0	35	6
3	2	27	20	35	17
4	3	24	0	40	13
5	2	29	8	38	15
6	1	25	20	36	1
7	3	24	23	32	14
8	3	25	14	32	28
9	2	24	4	31	12
10	3	26	12	31	18
11	2	24	2	33	28
12	1	27	23	31	29
13	2	29	2	34	22
14	1	23	1	33	8
15	1	24	8	34	14
16	1	30	10	32	12
17	3	27	12	37	26
18	2	29	27	39	12
19	2	30	17	31	20
20	1	28	6	36	4

## Data Availability

Not applicable.
